# Structural Color Colloidal Photonic Crystals for Biomedical Applications

**DOI:** 10.1002/advs.202403173

**Published:** 2024-07-31

**Authors:** Wenhui Zhang, Yangnan Hu, Pan Feng, Zhe Li, Hui Zhang, Bin Zhang, Dongyu Xu, Jieyu Qi, Huan Wang, Lei Xu, Zhou Li, Ming Xia, Jilai Li, Renjie Chai, Lei Tian

**Affiliations:** ^1^ School of Design and Arts Beijing Institute of Technology Beijing 100081 China; ^2^ State Key Laboratory of Digital Medical Engineering Department of Otolaryngology Head and Neck Surgery Zhongda Hospital School of Medicine Advanced Institute for Life and Health Jiangsu Province High‐Tech Key Laboratory for Bio‐Medical Research Southeast University Nanjing 210096 China; ^3^ Co‐Innovation Center of Neuroregeneration Nantong University Nantong 226001 China; ^4^ Department of Neurology Aerospace Center Hospital School of Life Science Beijing Institute of Technology Beijing 100081 China; ^5^ The Eighth Affiliated Hospital Sun Yat‐sen University Shenzhen 518033 China; ^6^ Department of Otolaryngology‐Head and Neck Surgery Shandong Provincial ENT Hospital Shandong University Jinan 250022 China; ^7^ Beijing Institute of Nanoenergy and Nanosystems Chinese Academy of Sciences Beijing 101400 China; ^8^ School of Nanoscience and Engineering University of Chinese Academy of Sciences Beijing 100049 China; ^9^ Department of Otolaryngology Shandong Provincial Hospital Affiliated to Shandong First Medical University Jinan 250021 China; ^10^ Medical Science and Technology Innovation Center Shandong First Medical University & Shandong Academy of Medical Sciences Jinan 250117 China; ^11^ Department of Neurology Aerospace Center Hospital Peking University Aerospace Clinical College Beijing 100049 China; ^12^ Department of Otolaryngology Head and Neck Surgery Sichuan Provincial People's Hospital School of Medicine University of Electronic Science and Technology of China Chengdu 610072 China; ^13^ Southeast University Shenzhen Research Institute Shenzhen 518063 China

**Keywords:** biosensors, cell research, drug delivery, photonic crystals, structural color

## Abstract

Photonic crystals are a new class of optical microstructure materials characterized by a dielectric constant that varies periodically with space and features a photonic bandgap. Inspired by natural photonic crystals such as butterfly scales, a series of artificial photonic crystals are developed for use in integrated photonic platforms, biosensing, communication, and other fields. Among them, colloidal photonic crystals (CPCs) have gained widespread attention due to their excellent optical properties and advantages, such as ease of preparation and functionalization. This work reviews the classification and self‐assembly principles of CPCs, details some of the latest biomedical applications of large‐area, high‐quality CPCs prepared using advanced self‐assembly methods, summarizes the existing challenges in CPC construction and application, and anticipates future development directions and optimization strategy. With further advancements, CPCs are expected to play a more critical role in biosensors, drug delivery, cell research, and other fields, bringing significant benefits to biomedical research and clinical practice.

## Graphical abstract

Colloidal photonic crystals (CPCs) have gained widespread attention due to their excellent optical properties and advantages. This work reviews the classification and self‐assembly principles of CPCs, and details some of the latest biomedical applications of large‐area, high‐quality CPCs prepared using advanced self‐assembly methods. Notably, the future perspectives and challenges associated with the biomedical applications of CPCs are discussed in depth.

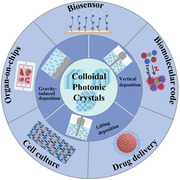

## 1 Introduction

**Figure 1:**
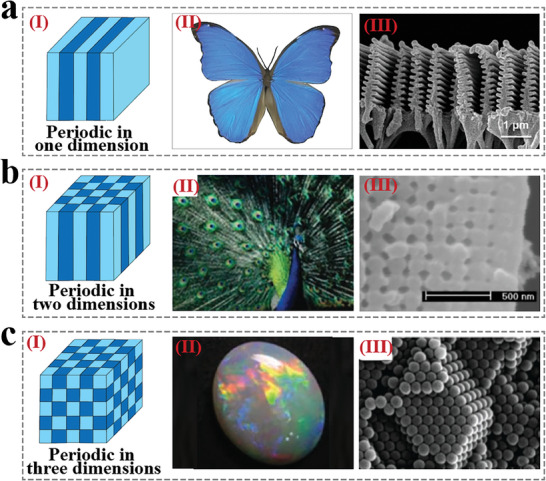
Typical PCs in natural creatures. a) Schematic representation of 1D CPC (I), photo (II), and SEM image (III) of *Morpho Menelaus* wings. *Morpho Menelaus* wing SEM image was reproduced with permission.^[^ ^16^ ^]^ Copyright 2017, The Authors. b) Schematic representation of 2D CPC (I), photo (II), and SEM image (III) of Peacock wings. The Peacock photo was reproduced with permission.^[^ ^17^ ^]^ Copyright 2010, Wily‐VCH. The Peacock wing SEM image was reproduced with permission.^[^ ^18^ ^]^ Copyright 2003, The National Academy of Sciences. c) Schematic representation of 3D CPC (I), photo (II), and SEM image (III) of Opals. Opal photo was reproduced with permission.^[^ ^17^ ^]^ Copyright 2010, Wiley‐VCH. Opal SEM image was reproduced with permission.^[^ ^5^ ^]^ Copyright 2009, Wiley‐VCH.

Photonic crystals (PCs) are one kind of photonic material with a spatially periodic variation in dielectric constant. They can generate a series of “forbidden” frequencies termed photonic bandgaps (PBGs).^[^ ^1^, ^2^, ^3^ ^]^ The light with certain wavelengths or frequencies that match the forbidden band cannot propagate through the PCs. When the wavelength of PBG falls within the visible light range, the PCs exhibit vibrant structural colors. Unlike pigments derived from chemical dyes, structural colors originate from the microscopic arrangement of materials and interference with the light of specific wavelengths, which are unaffected by chemical degradation and possess a metallic luster.^[^ ^4^ ^]^ PCs with various colors are widespread in nature, such as the wings of the *Morpho* butterfly, peacock feathers, and opals.^[^ ^5^, ^6^, ^7^, ^8^ ^]^ PC materials can be divided into 1D, 2D, and 3D PCs based on the number of dimensions in the PBG,^[^ ^4^ ^]^ as presented in **Figure** **1**. In recent years, with the revelation of the optical and structural mechanisms of these natural PCs, researchers have designed and developed various intelligent materials based on PCs from a biomimicry perspective.

Colloidal photonic crystals (CPCs) are PCs prepared using a “bottom‐up” approach by employing monodisperse colloidal particles (small particles ranging from nanometers to micrometers in diameter).^[^ ^9^, ^10^, ^11^, ^12^ ^]^ Their unique structure, vibrant structural colors, customizable functionalities, and ease of construction make them ideal functional materials. CPCs with specific functions can be obtained by selecting suitable particles and matrices for preparation.^[^ ^13^, ^14^, ^15^ ^]^ In recent years, owing to their outstanding properties, CPCs have gained significant attention and applications in biomedicine, particularly in biosensors, drug delivery, cell research, and other areas.

**Figure 2:**
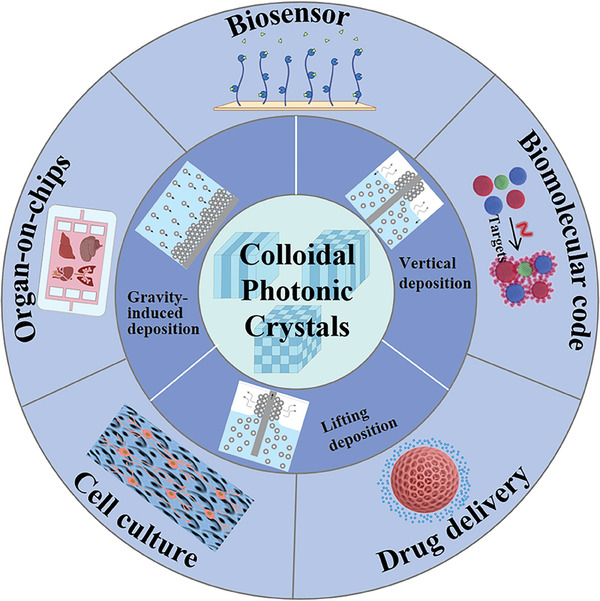
Schematic illustration of the fabrication methods and biomedical applications of CPCs.

Herein, we present the latest research advancements of CPCs in biomedicine. The fundamental building blocks of CPCs, including colloidal particles and the polymeric matrix, are first introduced. Afterward, the primary design types of CPCs widely employed in the biomedical field, such as close‐packed, non‐close‐packed, and inverse opal PCs, are outlined (**Figure** **2**). Notably, the significant applications of CPCs in various areas, such as early diagnosis of cancer, biomolecular coding, biosensors, cell culture, and drug delivery, are then emphasized. Finally, the future perspectives and challenges associated with the biomedical applications of CPCs are further discussed.

## 2 Building Blocks for CPCs

CPCs are composed of colloidal particles with appropriate size and are often combined with polymer matrices to acquire more functions and responsiveness. The optical properties of CPCs are determined by the periodic arrangement and refractive index (RI) of the colloidal particles as well as the surrounding medium. Briefly, the periodic arrangement is the organized and repeating structure of colloidal particles that form a crystal lattice. This arrangement leads to the formation of photonic bandgaps, where certain wavelengths of light cannot propagate through the structure. The RI indicates how light propagates through those materials. Therefore, the selection of colloidal particles and polymer matrix is crucial for building functional CPCs. Herein, we introduce the common colloidal particles, including inorganic, organic, and organic–inorganic hybrid colloidal particles, and the polymeric matrices with different capabilities, such as shape change, healing, and optical property changes in building CPCs.

### 2.1 Colloidal Particles

#### 2.1.1 Inorganic Colloidal Particles

Inorganic materials (typically minerals or metals) are widely used for constructing CPCs because of their stability, high‐temperature resistance, and compatibility. Silica (SiO_2_) is the preferred inorganic material for fabricating CPCs ascribed to its low loss rate and high RI.^[^ ^19^, ^20^, ^21^ ^]^ Currently, strategies for preparing SiO_2_ CPCs include gravity‐induced deposition, vertical deposition, lifting deposition, etc.^[^ ^10^, ^22^, ^23^, ^24^ ^]^ The fabrication methods will be detailed in the next section. In addition to SiO_2_, copper and its oxides have also attracted extensive attention for the fabrication of CPCs due to their simple and easy‐to‐access properties.^[^ ^25^ ^]^ For instance, cuprous oxide (Cu_2_O) is a semiconductor material with unique optical and magnetic properties, based on which CPC with an RI as high as 2.7 has been prepared.^[^ ^26^ ^]^ The fabricating approaches for Cu_2_O particles include electrolysis, hydrothermal, and chemical precipitation, etc.^[^ ^27^, ^28^, ^29^ ^]^ Inorganic particles such as cerium dioxide, zinc sulfide, cadmium sulfide, and Fe_3_O_4_ nanoparticles have also been utilized in CPC preparation.^[^ ^30^, ^31^, ^32^, ^33^, ^34^ ^]^

#### 2.1.2 Organic Colloidal Particles

Benefitting from simple preparation procedures, low cost, and easy chemical modification, organic materials have also been employed to construct CPCs. The most commonly utilized organic materials for preparing CPCs are polystyrene (PS) and polymethyl methacrylate (PMMA).^[^ ^35^, ^36^, ^37^, ^38^ ^]^ PS particles can form an ordered face‐centered cubic structure through the self‐assembly process, allowing for the adjustment of the photonic gap position by altering PS particles' radius, which enables the regulation of optical properties. In addition, PS particles can be combined with other components to modulate the optical properties of resulting CPCs. For example, Wu et al. covalently combined amine‐rich carbon dots (CDs) with carboxyl‐rich poly (styrene‐acrylic acid) (P (St‐AA)) particles to form a large‐scale CPC.^[^ ^39^ ^]^ Besides, they integrated CDs‐grafted P (St‐AA) colloidal particles with supramolecular hydrogels to produce CPC hyperspherical hydrogels, effectively leveraging the properties of both. Moreover, PMMA particles can also be utilized as the basic structural unit of CPCs. For example, Kassim et al. successfully prepared CPCs using Au nanoparticle‐coated PMMA particles generated from emulsion polymerization.^[^ ^40^ ^]^ In another study, Wang et al. used emulsion polymerization to obtain degradable PMMA colloidal nanoparticles with high‐quality fractions (30 wt%) and further prepared PMMA liquid CPCs. The corresponding precrystallization forms were effectively prepared using the rotating evaporation/surfactant method, which ensures the structural stability and color saturation consistency of PMMA CPCs.^[^ ^41^ ^]^

#### 2.1.3 Organic–Inorganic Hybrid Colloidal Particles

Organic–inorganic hybrid colloidal particles are composed of both organic and inorganic components. They possess the flexibility and processability of organic particles, as well as the thermal stability and mechanical strength of inorganic particles, making them versatile and applicable in various fields. For example, Li et al. used a simple two‐step chemical method to synthesize composite Fe_3_O_4_@PS colloidal particles. These particles exhibited excellent response behavior to electric and magnetic fields in the suspended solution, and the resultant CPCs displayed high reflectivity and structural color.^[^ ^42^ ^]^ By crosslinking polymer single‐chain tethered onto Fe_3_O_4_@SiO_2_ nanoparticles, Yang et al. described a method for electrostatic‐mediated synthesis of reactive Janus inorganic/polymer colloidal dimers. The amphiphilic dimer synthesized by this method could be effectively manipulated by magnets to generate stable milk droplets and could be further modified to obtain a series of functional dimers.^[^ ^43^ ^]^

### 2.2 Polymeric Matrices

Polymers are essential in constructing the structure of CPCs, and their selection and design significantly impact the performance and functionality of CPCs. The polymer matrix is considered as the continuous phase in the photonic crystal structure, providing stability and support to the overall structure by connecting adjacent colloidal particles.^[^ ^44^ ^]^ The characteristics of this continuous phase are crucial for the optical properties of CPCs. The careful selection of the components and structure of the polymer matrices allows the modulation of the periodic structure of CPCs, thereby influencing their reflective and transmissive properties. Additionally, different polymer matrices can provide CPCs with deformability, self‐healing capability, and specific wetting properties. Furthermore, some polymers with unique properties can impart stimulus‐responsive characteristics to CPCs, showcasing significant potential in applications such as visual sensing, detection, anti‐counterfeiting, and more.^[^ ^45^, ^46^ ^]^ Therefore, according to the different properties, the polymer matrices applied as CPCs were separated into polymeric matrices with shape‐change, healable, and optical property‐change capabilities (**Table** **1**).

**Table 1:** Classification of polymeric matrices for the construction of CPCs.

Polymeric matrices	Mechanism	Refs.
Polymeric matrices with shape‐change capabilities	Relaxation phenomena, non‐uniform strain, the affinity between polymer and solvent, interchain interaction, crosslink density of the polymer	[47, 48, 49, 50]
Polymeric matrices with healable capabilities	Dynamic covalent bonds (such as imine bonds, borate bonds, disulfide bonds, etc.), physical interactions (such as hydrophobic interactions, hydrogen bonds, π–π stacking, etc.)	[51, 52, 53, 54]
Polymeric matrices based on optical property changes	Refractive index, optical transmittance	[45, 55, 56]

#### 2.2.1 Polymeric Matrices with Shape‐Change Capabilities

Polymeric matrices possessing shape‐changing capabilities can undergo alterations in their macroscopic shape when subjected to specific external stimuli.^[^ ^57^ ^]^ This property allows the periodic structure of CPCs to undergo changes under external stimuli, thereby causing structural color variations.^[^ ^58^, ^59^ ^]^ The most effective method to induce shape changes in polymer matrices is applying stress to deformable materials. Flexible hydrogels and elastomers are commonly used as deformable matrices. Additionally, temperature, light, or electric fields can induce macroscopic shape alterations in polymer matrices through the relaxation behavior of polymer chains or non‐uniform strain, thereby adjusting the periodic structure of CPCs. Shape memory materials are a class of special materials that can “remember” their original shape and restore the original shape under certain conditions.^[^ ^47^, ^60^ ^]^ Shape memory polymers are available in various types, including epoxy‐based, cyanate ester‐based, polyimide‐based, and PS‐based shape memory polymers.^[^ ^61^, ^62^, ^63^ ^]^ They mainly have shape memory effect, super elasticity, good recovery accuracy, fatigue resistance, response, stability, designability, and so on. Such materials usually have a specific microstructure inside them, such as having different phase states. In the initial state, the material is in a more stable phase (called the parent phase). When subjected to external stimuli, such as temperature changes, the material will undergo a phase transition, transforming into another phase (called the martensitic phase), which can be deformed more easily. When the external stimulus changes again, such as when the temperature returns to the original level, the material will undergo a reverse phase transition, from martensitic phase transition back to the parent phase, and thus return to the original shape.^[^ ^64^ ^]^ Overall, these polymers with shape‐changing capabilities play a multifaceted role in constructing CPCs, ranging from shape tuning to deformability and optical responsiveness, providing CPCs with extensive functionality and application potential. For example, the structure of CPCs can be controlled by the characteristics of shape memory material to realize the dynamic control of light propagation, reflection, and refraction. By changing the shape of the shape memory material, the optical bandgap of the CPCs can be adjusted to achieve the function of switching or modulating light on demand.

In addition, volume changes in polymer matrices can alter the lattice spacing of polymers, leading to variations in structural color.^[^ ^65^ ^]^ For example, thermoresponsive polymers can change their volume when subjected to different external temperatures. Poly(N‐isopropylacrylamide) (pNIPAM) is one of the most extensively studied thermoresponsive polymers, whose thermoresponsiveness is reflected in the observed hydrophobic‐hydrophilic phase transition in aqueous solution upon heating. pNIPAM exhibits a lower critical solution temperature (LCST) of 32 °C and has gained widespread attention and research in tissue engineering, sensors, and drug delivery.^[^ ^50^, ^66^, ^67^, ^68^ ^]^ When the temperature falls below its LCST, hydrogen bonds form between the amide groups and water, rendering pNIPAM water‐soluble and adopting an expanded coiled conformation. If the external temperature exceeds the LCST, the hydrogen bonds weaken, and hydrophobic interactions between isopropyl groups and the polymer backbone become stronger. Water molecules are squeezed out of the polymer, causing pNIPAM to adopt a spherical structure.

Light‐responsive polymers adjust their macroscopic volume by modulating the affinity between the polymer and solvent through light stimulation.^[^ ^69^ ^]^ The light‐induced changes in photosensitive polymers are achieved by photochemical reactions of introduced photosensitive molecules, such as spiropyran, triphenylmethane, and azobenzene.^[^ ^70^ ^]^ Additionally, pH‐responsive polymers rely on external pH changes to modulate their interchain interactions and alter the volume of the polymer matrix. A distinctive structural characteristic of pH‐responsive polymers is the presence of ionizable groups capable of accepting and releasing protons under varying environmental pH conditions. It is worth mentioning that the changes in polymer matrix volume induced by protonation and deprotonation of pH‐responsive polymers also involve changes in the affinity between the polymer and the solvent.^[^ ^71^ ^]^ Moreover, the crosslinking density of polymers also contributes to alterations in the volume of the polymer matrix. For example, in hydrogels, certain stimuli, such as ions and small molecules, can induce additional crosslinking, consequently altering their volume. For instance, hydrogels containing phosphate groups can form a secondary network when immersed in an iron ion solution due to the strong complexation between the ferric and phosphate groups, causing a notable shrinkage.^[^ ^72^ ^]^

#### 2.2.2 Polymeric Matrices with Healable Capabilities

Endowing materials the ability to self‐heal and regain their original properties is crucial for improving material performance and extending their lifespan, opening up new prospects for more sustainable technologies. Polymeric matrices with self‐healing capabilities refer to a class of polymers that can connect the segments to reform a bulk. The inherent self‐healing ability of these materials is typically achieved through the rearrangement of reversible bonds, including dynamic covalent bonds (such as imine bonds, borate bonds, disulfide bonds, etc.) and physical interactions (such as hydrophobic interactions, hydrogen bonds, π–π stacking, etc.).^[^ ^51^, ^52^, ^53^ ^]^ Upon experiencing fractures, the dissociation of dynamic crosslinking points facilitates the migration and diffusion of polymer chains in the vicinity of the damaged area. These chains can re‐establish bonding interactions, thereby achieving self‐repair on a macroscopic scale.^[^ ^54^ ^]^

#### 2.2.3 Polymeric Matrices Based on Optical Property Changes

Some polymers can undergo physical and chemical changes when exposed to external stimuli, leading to changes in RI.^[^ ^45^, ^55^ ^]^ This variation can alter the RI of CPC and RI contrast, thereby inducing a shift in structural color without modifying the lattice spacing. In addition, external stimuli can also cause changes in the light transmittance of certain polymer matrices, dynamically concealing the color of the internal photonic structure, thereby achieving a transition from structural color to the intrinsic optical signal of the polymer matrices. As an illustration, polyethylene glycol (PEG) exhibits an optical transmittance close to zero in its crystalline state, resulting in a milky white appearance. Conversely, in the molten state, the optical transmittance of PEG surpasses 85%, rendering it nearly fully transparent.^[^ ^56^ ^]^

## 3 The Fabrication of CPCs with Diverse Nanostructures and Shapes

Currently, the methods for preparing CPCs are mainly categorized into “top‐down” and “bottom‐up” techniques, which leads to various nanostructures and shapes of CPCs. The “top‐down” strategy is typically based on microfabrication techniques such as photolithography and ion etching to carve solid materials into micro‐nanostructures and form periodically ordered arrays. Although the “top‐down” method can produce CPCs with excellent morphology and size, the complex production process and expensive manufacturing costs limit its practical applications. In contrast, the “bottom‐up” method involves the self‐assembly of materials to form ordered periodic structures. This strategy is straightforward, cost‐effective, and efficient, establishing it as the predominant method for fabricating CPCs. According to the nanostructures, CPCs can be classified into three major types: close‐packed CPCs, non‐close‐packed CPCs, and inverse opal CPCs. These CPCs can be shaped into various forms, such as films, blocks, microspheres, patterns, etc., to meet multiple application requirements. In this section, we briefly outline the design and manufacturing strategies for these CPCs.

### 3.1 Close‐Packed CPCs

**Figure 3:**
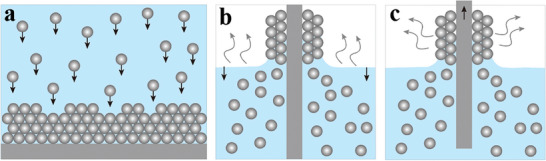
Scheme of fabrication methods for close‐packed CPCs. a) Gravity‐induced deposition. b) Vertical deposition. c) Lifting deposition.

The close‐packed CPCs refer to highly ordered arrays of closely packed colloidal particles. Various methods for preparing close‐packed CPCs have been reported, including gravity‐induced deposition, vertical deposition, and lifting deposition.^[^ ^4^, ^73^, ^74^ ^]^ Among them, gravity‐induced deposition is the simplest method, where monodisperse colloidal particles naturally settle under gravity to form a hexagonal arrangement (**Figure** **3a**). However, this method is time‐consuming and challenging to control regarding the morphology of the prepared CPCs, and particles that are too small or too large face obstacles in self‐assembling through gravity‐driven deposition. On the basis of gravity‐driven self‐assembly, applying external fields, such as light, heat, electricity, magnetism, and mechanical force, allows for the ordered assembly of particles. Among them, magnetic induction emerges as a powerful tool due to its flexibility, adjustability, noncontact manner, and rapid response. For instance, Chen et al. proposed a strategy of magnetic‐induced self‐assembly to produce an ordered close‐packed lattice structure composed of Fe_3_O_4_@SiO_2_ nanoparticles, exhibiting vibrant structural colors and serving as encoding labels for biological detection.^[^ ^75^ ^]^ In addition, vertical deposition stands out as one of the most effective methods for producing high‐quality CPCs, such as large‐scale SiO_2_ films and inverse‐SiO_2_ PMMA hydrogel films.^[^ ^76^, ^77^ ^]^ During vertical deposition, the meniscus level continuously moves downward due to the solvent evaporation from the colloidal solution (Figure 3b). Deposition initiates with the pinning of colloidal nanoparticles at the menisci region, followed by the movement of other nanoparticles toward the pinned ones driven by the flow of evaporating solution. Subsequently, monodisperse colloidal particles assemble into an ordered structure due to capillary force. In particular, precise control of film thickness can be achieved by manipulating the solvent evaporation rate and particle concentration. Following optimization, researchers proposed the “lifting deposition” method for producing high‐quality CPCs to reduce liquid evaporation duration.^[^ ^78^ ^]^ As depicted in Figure 3c, the substrate is lifted at a constant speed, obviating the dependence on solvent evaporation. By precisely controlling the lifting speed and the concentration of particles, the film thickness can be customized from a monolayer to several tens of layers.

**Figure 4:**
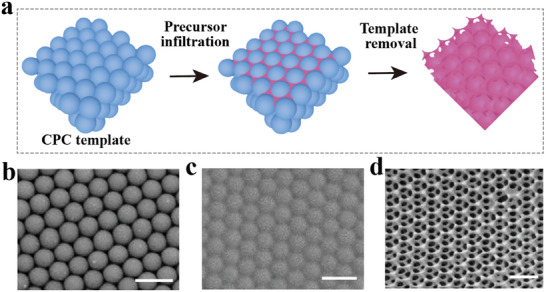
Preparation methods for inverse opal PCs. a) Fabrication process of the inverse opal using template removal method. b–d) SEM images of CPC template (b), hydrogel‐infiltrated CPC (c), and inverse opal‐structured hydrogel (d). Reproduced with permission.^[^ ^83^ ^]^ Copyright 2020, The Authors, published by American Association for the Advancement of Science.

Inverse opal is a negative replication product of close‐packed CPC templates. In 1997, inverse opal was first developed via protein‐liposome interactions by Velev.^[^ ^79^ ^]^ They assembled PS particles into a cubic close‐packed lattice and replicated such a structure via the deposition in the interstitial spaces between the particles, followed by the selective removal of the PS template by calcination. Since then, various inverse opals prepared from different materials have been developed and reported.^[^ ^68^, ^80^, ^81^, ^82^ ^]^ **Figure** **4a** illustrates the classical method for preparing inverse opal structures. Specifically, a curable precursor material is filled into the interstitial spaces among colloidal particles. After that, the CPC template is removed through calcination, chemical etching, or solvent dissolution, finally leaving a periodically porous structure with a high surface‐to‐volume ratio. Figure 4b–d depicts the scanning electron microscope (SEM) images of the CPC template, infiltrated CPC, and the obtained inverse opals, respectively. SiO_2_ and polymer particles are the most common colloidal particles used for template preparation due to their excellent monodispersity and ease of removal. SiO_2_ can be removed through hydrofluoric acid etching, while polymers can be eliminated through suitable solvents, calcination, or thermal decomposition. Some polymers that can undergo solidification can serve as the scaffold materials for inverse opals.

### 3.2 Non‐Close‐Packed CPCs

**Figure 5:**
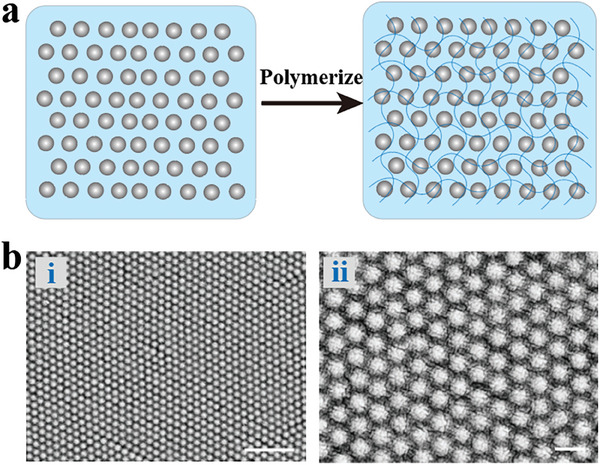
Preparation and structure of non‐close‐packed CPCs. a) Scheme illustrating the preparation of non‐close‐packed CPCs. b,c) SEM image of the non‐close‐packed CPCs. Scales bars are 1 µm in (i) and 200 nm in (ii). Reproduced with permission.^[^ ^85^ ^]^ Copyright 2022, The Authors, published by Wiley‐VCH.

Dispersing charged colloidal particles in a polar medium like water can promote their assembly into crystal structures to minimize electrostatic repulsion interactions. At high concentrations of colloidal particles, significant inter‐particle repulsion force occurs, leading the particles (such as poly[styrene‐co‐(sodium 1‐allyloxy‐2‐hydroxypropanesulfonate)] latex particles) to self‐assemble into non‐close‐packed colloidal crystal arrays (**Figure** **5a**).^[^ ^84^ ^]^ The SEM images in Figure 5b displayed the structure of non‐close‐packed silica nanoparticles. However, these non‐close‐packed arrangements are easily disrupted by exterior stimuli, such as external shock or ion impurities. Introducing nonionic polymerizable monomers into the system can lock the ordered particles in a hydrogel matrix through the crosslink of these monomers. The lattice order of structures prepared by this method no longer depends on the electrostatic interactions between particles, thus generating more stable polymerized colloidal crystal arrays. Additionally, by selecting different monomers, different properties can be imparted to CPCs to meet various application requirements.

### 3.3 Tuning of Structural Colors

The nanostructures (close‐packed and non‐close‐packed) of CPCs affect their structural colors significantly. In fact, tuning the structural colors of CPCs involves several methods that modify the photonic bandgap, affecting the wavelengths of light that are reflected or transmitted.^[^ ^86^ ^]^ The methods include: 1) Adjusting the particle size: Changing the diameter of the colloidal particles alters the photonic bandgap. Larger particles shift the structural color toward longer wavelengths (red), while smaller particles shift it toward shorter wavelengths (blue). For example, the structural color of close‐packed spherical silica nanoparticles depends on their size, affecting the reflected wavelengths. Typically, silica nanoparticles sized 180– 500 nm produce violet to red colors. 2) Changing the RI contrast: Modifying the materials used for the colloidal particles or the surrounding medium can change the RI contrast, thereby affecting the structural color. Higher RI contrast typically enhances the intensity of the structural colors. 3) Controlling the lattice spacing (close‐packed and non‐close‐packed): The spacing between the colloidal particles can be adjusted by varying the concentration of the colloidal suspension or by applying external forces (e.g., electric or magnetic fields). This changes the periodicity of the structure and, consequently, the reflected wavelengths. 4) Temperature and solvent effects: Temperature changes can cause thermal expansion or contraction of the colloidal particles, affecting their size and the lattice spacing. Similarly, swelling or shrinking the particles by changing the solvent can tune the structural colors. 5) Incorporation with other materials: Introducing other materials, such as dyes or nanoparticles, can modify the optical properties of the colloidal photonic crystals, thereby altering the structural colors. 6) Applying external mechanical strain: Mechanically stretching or compressing the photonic crystal structure can change the lattice parameters, thus tuning the photonic bandgap and the structural color. 7) Electrostatic or magnetic control: Applying electric or magnetic fields can manipulate the alignment and spacing of the colloidal particles, providing a dynamic way to tune the structural colors. By employing these methods, it is possible to precisely control the structural colors of colloidal photonic crystals for various biomedical applications.

## 4 Biomedical Application of CPCs

CPCs formed by the periodic arrangement of media with different refractive indices can regulate the propagation and absorption characteristics of light by changing the refractive index, pore size, and arrangement of the medium. Ascribed to their ability to manipulate light, CPCs have become a powerful optical material widely used in optics, sensors, optoelectronics, and other fields. Nowadays, CPCs have also attracted increasing attention in biomedicine, including but not limited to biosensors, drug delivery, and cell research (**Table** **2**).^[^ ^12^, ^87^ ^]^

**Table 2:** A summary of CPCs for biomedical applications.

Applications	Colloidal particles	Matrix	Morphology	Purpose	Refs.
Biosensors	Polystyrene nanoparticles	polyacrylamide (PAM)	Film	Competition‐based CPC hydrogel biosensors capable of naked‐eye detection of various biomolecules (e.g., proteins, peptides, and small molecules)	[88]
	PMMA microspheres	/	Film	Enhanced up‐conversion fluorescence sensor of COVID‐19 antibody	[89]
	Silica nanoparticles	Composite hydrogel containing acrylic acid, chitosan, and carbon nanotube	Film	Wearable strain and temperature sensor	[90]
Biomolecular code	Silica nanoparticles	/	Sphere	Multiplex, high‐selectivity, and high‐sensitivity miRNAs screening	[91]
	Silica nanoparticles	Composite hydrogel containing Poly(ethylene glycol) diacrylate (PEGDA), PEG, and acrylic acid	Sphere	Multiplex miRNA quantification	[92]
	Silica nanoparticles	/	Sphere	Multiplex exosome capturing and screening	[93]
Drug delivery	Fe_3_O_4_@C nanoparticles	/	Chain	Synchronous on‐demand drug release and visual real‐time monitoring of drug content	[94]
	SiO_2_ nanoparticles	PEGDA	Microneedle	Loading various drugs and monitoring drug release	[95]
	Silica nanoparticles	Methylacrylylated silk fibroin	Sphere	Local inner ear drug delivery	[96]
Cell culture	Silica nanoparticles	PS	Film	Substrates for PC12 cell culture and orientation	[97]
	Silica nanoparticles	PS	Film	Substrates for neural stem cell culture and orientation	[98]
Organ‐on‐a‐chip	Silica nanoparticles	GelMA	Film	Heart‐on‐a‐chip platform for biological research and drug screening	[99]
	Silica nanoparticles	GelMA	Film	Cardiac fibrosis‐on‐a‐chip providing microphysiological visuals	[100]
	Silica nanoparticles decorated with negative charge	Acrylamide	Film	Heart‐on‐a‐chip for Cardiomyocyte sensing and drug evaluation	[85]

### 4.1 Biosensors

**Figure 6:**
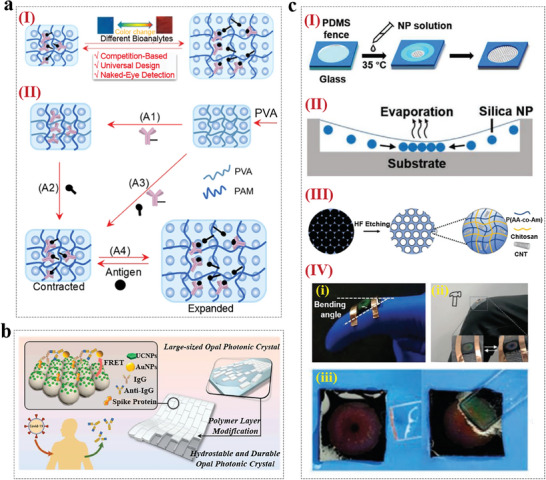
CPCs for biosensors. a) (I) The principle of the biosensor based on the antigen‐antibody interaction. (II) Schematic of the detection process of this competitive biosensor. Reproduced with permission.^[^ ^88^ ^]^ Copyright 2020, American Chemical Society. b) Schematic diagram of the operation of the upconversion‐photonic crystal fluorescence sensor. Reproduced with permission.^[^ ^89^ ^]^ Copyright 2023, Elsevier. c) Scheme of the fabrication process (I) and mechanism (II) of CPCs by horizontal precipitation, scheme of the fabrication process of CPC hydrogel (III), and monitoring of human motions including finger bending by wearable CPC hydrogel sensors: finger bending (i), knee‐jerk reaction (ii), and pressure (iii). Reproduced with permission.^[^ ^90^ ^]^ Copyright 2022, Wiley‐VCH.

Biosensors are a class of analytical devices that can specifically recognize biomolecules or biomolecular interactions and convert into an equal amount of visible or readable signals for quantitative or qualitative analyses of the target, which are widely used in the fields of oncology detection, microbiology detection, and so on.^[^ ^101^, ^102^, ^103^ ^]^ As a special optical material, CPC has excellent optical properties and specific diffraction wavelengths. When CPCs respond to physical, chemical, or biological stimuli, their diffraction peaks change due to the changes in lattice spacing, arrangement, or RI of the surrounding area, and therefore, have gradually been used in the field of biosensors to detect the onset and development of various diseases.^[^ ^104^, ^105^, ^106^, ^107^, ^108^ ^]^ For instance, Qin et al. presented a competition‐based photonic crystal hydrogel biosensor capable of detecting various biomolecules such as proteins, peptides, and small molecules through antigen‐antibody interactions (**Figure** **6a**).^[^ ^88^ ^]^ The sensor was fabricated by doping photonic crystal particles in polyvinyl alcohol and chemically modifying antigens and antibodies during hydrogel polymerization. When a free antigen is present, antibodies immobilized in the hydrogel specifically recognize it, rupturing the hydrogel and causing a significant but reversible expansion. As a result, the blue or red light from the crystalline plane is displaced, and the color change can be captured by the naked eye or smartphone and used to evaluate the concentration and properties of the analyte. The sensor had a large degree of wavelength variation in binding analytes, exhibited high sensitivity, low background noise, and no dependence on the size, charge, or other properties of the target, but only on the antigen‐antibody interaction. This feature greatly improved the performance and versatility of the sensor, making it possible to detect proteins, peptides, and small molecules with high sensitivity and specificity, and will be synchronized to detect a wide range of analytes. This new type of photonic crystal biosensor has excellent potential for future development in disease diagnosis. Recently, the rapid and accurate detection of the new coronavirus has become a significant challenge due to the global emergence of the new coronavirus pneumonia outbreak, which has a rapid spread rate and high mutation rate, and has posed a danger to the family community. For more sensitive detection of novel coronaviruses, Hu et al. developed a novel and highly stable upconversion fluorescence resonance energy transfer (FRET) sensitive sensor (Figure 6b).^[^ ^89^ ^]^ The fluorescence intensity of upconverted nanomaterials was enhanced by exploiting the photonic crystal effect, resulting in more sensitive detection and good performance for detecting antibodies to novel coronaviruses with a detection limit of 0.1 ng mL^−1^. This study will provide a promising and effective tool in view of the increasing demand for early detection of novel coronaviruses.

Wearable sensors are devices that continuously and real‐time diagnose or monitor human health conditions.^[^ ^109^, ^110^, ^111^ ^]^ These devices are typically designed to be attached to the skin or other body parts and have the ability to convert human activity into electronic or optical signals. Routine changes, such as electrocardiograms, blood pressure, blood glucose, etc., can be monitored using non‐invasive wearable sensors. Wearable devices typically use soft materials such as elastomers and hydrogels as the backbone, while carbon nanotubes, graphene, conductive polymers, and other functional materials are often used as additives to give the device more functional properties.^[^ ^112^ ^]^ However, signal readout often requires expensive devices. Visual changes integrated directly into wearable devices provide a new strategy for intuitively accessing the response signals of wearable devices. The structural color of CPCs has attracted new attention in wearable sensors.^[^ ^90^, ^113^ ^]^ Liu et al. developed a structured color hydrogel sensor with rapid response to different stimuli such as stress and temperature by using self‐assembly of silica particles and infiltrating a composite hydrogel containing materials such as acrylic acid, chitosan, and carbon nanotubes into the voids among the silica particles to form an inverse opal photonic crystal sensor (Figure 6c).^[^ ^90^ ^]^ Owing to the incorporation of carbon nanotubes, the composite hydrogel could output an electronic signal simultaneously when a color change occurs. Based on these properties, a CPC hydrogel sensor was formed for quantitatively monitoring external stimuli such as stretching, squeezing, and thermal stimuli. The authors suggested different applications of the obtained wearable sensors for monitoring the tension or compression during joint motion. Rapidly self‐assembling CPC hydrogels hold significant promise for use in wearable strain and temperature sensors. To fulfill the demands of personalized and preventive medical applications, these devices should be further enhanced for stretchability, high sensitivity, and light weight.

In addition, CPCs have great potential in multiplex detection and coding and have received extensive attention and research.^[^ ^92^, ^114^, ^115^, ^116^ ^]^ Universal, sensitive, and real‐time biomolecular analysis is essential for understanding the complex regulatory relationships between signaling, metabolism, and other biochemical activities in cells for disease diagnosis.^[^ ^117^ ^]^ miRNA is a non‐coding single‐stranded RNA molecule consisting of ≈22 nucleotides involved in the post‐transcriptional regulation of gene expression in organisms.^[^ ^118^ ^]^ miRNAs are often closely associated with tumorigenesis, and therefore, their abnormal expression in vivo can serve as highly accurate markers for tumor diagnosis.^[^ ^119^, ^120^, ^121^ ^]^ Since the content of miRNA in the blood is extremely low and quickly degrades, quantitative detection of miRNAs is difficult and challenging for current tumor diagnosis.^[^ ^122^ ^]^ Periodic amplification on microarray chips is a common method for quantitative miRNA analysis. Still, it is limited to only single‐target detection due to the immaturity of the technology and high equipment cost, which is not applicable to high‐throughput analysis and low‐concentration detection.^[^ ^123^, ^124^, ^125^ ^]^ In order to meet the multiplexed analysis need with high sensitivity and throughput, photonic crystal barcodes are gradually being developed for the sensitive detection of multiple biomolecules.^[^ ^126^, ^127^, ^128^, ^129^ ^]^

**Figure 7:**
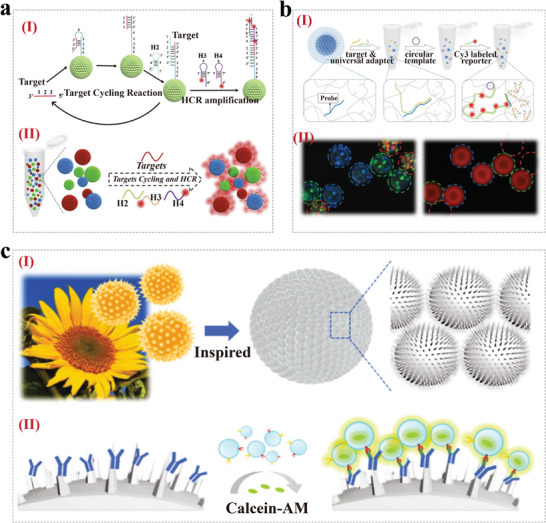
CPCs for biomolecular code. a) (I) Schematic diagram of the detection mechanism of the photonic crystal barcode. (II) Schematic representation of the barcodes modified with different probes to detect multiple miRNAs. Reproduced with permission.^[^ ^91^ ^]^ Copyright 2019, Elsevier. b) (I) Schematic diagram of RCA and photonic crystal integrated barcode operation. (II) Optical and fluorescent images of three differently modified barcodes after incubation with miRNAs. Reproduced with permission.^[^ ^92^ ^]^ Copyright 2017, Wiley‐VCH. c) (I) Morphology of sunflower pollens and the inspired CPC barcodes. (II) Scheme of specific recognition of pollen‐inspired barcodes in exosome capturing and screening. Reproduced with permission.^[^ ^93^ ^]^ Copyright 2022, The authors.

For example, Zhao et al. developed a novel barcode by integrating a hybridization by hybridization amplification (HCR) approach into polydopamine (PDA)‐modified photonic crystal particles (**Figure** **7a**).^[^ ^91^ ^]^ In this work, PDA‐encapsulated SiO_2_ CPCs were combined with multiple probe molecules to achieve multiple detection of miRNAs. The amplification cycle was started when the target miRNA was present, and the fluorescence intensity was positively correlated with the concentration of the target miRNA. The final amplification product was quantitatively detected by measuring the fluorescence signal of the CPCs. The obtained barcode showed high sensitivity and specificity, providing a promising multiplexed detection platform for clinical detection. In addition, Xu et al. presented a barcode for detecting low‐abundance miRNAs by combining rolled circular amplification (RCA) with CPCs, which enabled high‐throughput analysis (Figure 7b).^[^ ^92^ ^]^ RCA is an isothermal nucleic acid amplification method and targeted molecule spotting strategy with high fidelity, specificity, and sensitivity. The porous hydrogel‐encapsulated CPC barcodes were synthesized by filling pregel into the voids between the assembled SiO_2_ particles. The inverse opal structure of the porous hydrogel was interconnected with the hydrophilic scaffold, which could provide a homogeneous surrounding medium for miRNA targeting reactions and RCAs. Integration of the advantages of photonic crystal barcoding and RCA enables rapid quantification of low‐abundance miRNA with a lower limit of detection as low as 20 fm, providing strong sensitivity and specificity. Inspired by the structure of pollens, Li et al. fabricated CPC barcodes with prickly surfaces for multiplex capturing and screening of exosomes (Figure 7c).^[^ ^93^ ^]^ Figure 7c‐I shows the scheme of the design and application of the pollen‐inspired CPC barcode assembled by surface prickly microspheres with a pollen‐like structure. By modifying the CPC barcodes with antibodies, exosomes could be efficiently captured and screened by specific recognition on the barcodes, as well as highly sensitive multiplex detection (Figure 7c‐II). In summary, CPC barcodes have become reliable tools for multiplexed detection due to the characteristic reflection peaks generated by their periodic structure, and they play an important role in biomedical applications that require multiplexing.

### 4.2 Drug Delivery

The most common modes of administration currently include intravenous, intramuscular, and subcutaneous injections. Regardless of the mode of injection, many drugs are rapidly cleared from the body and are uncontrollable in terms of dose and speed of administration. Moreover, there is even a risk of disease transmission due to the reuse of injection needles. Therefore, controlled drug delivery systems can improve drug bioavailability, therapeutic efficacy, and safety and play a vital role in medicine and healthcare.^[^ ^130^, ^131^, ^132^, ^133^, ^134^, ^135^, ^136^, ^137^, ^138^ ^]^

**Figure 8:**
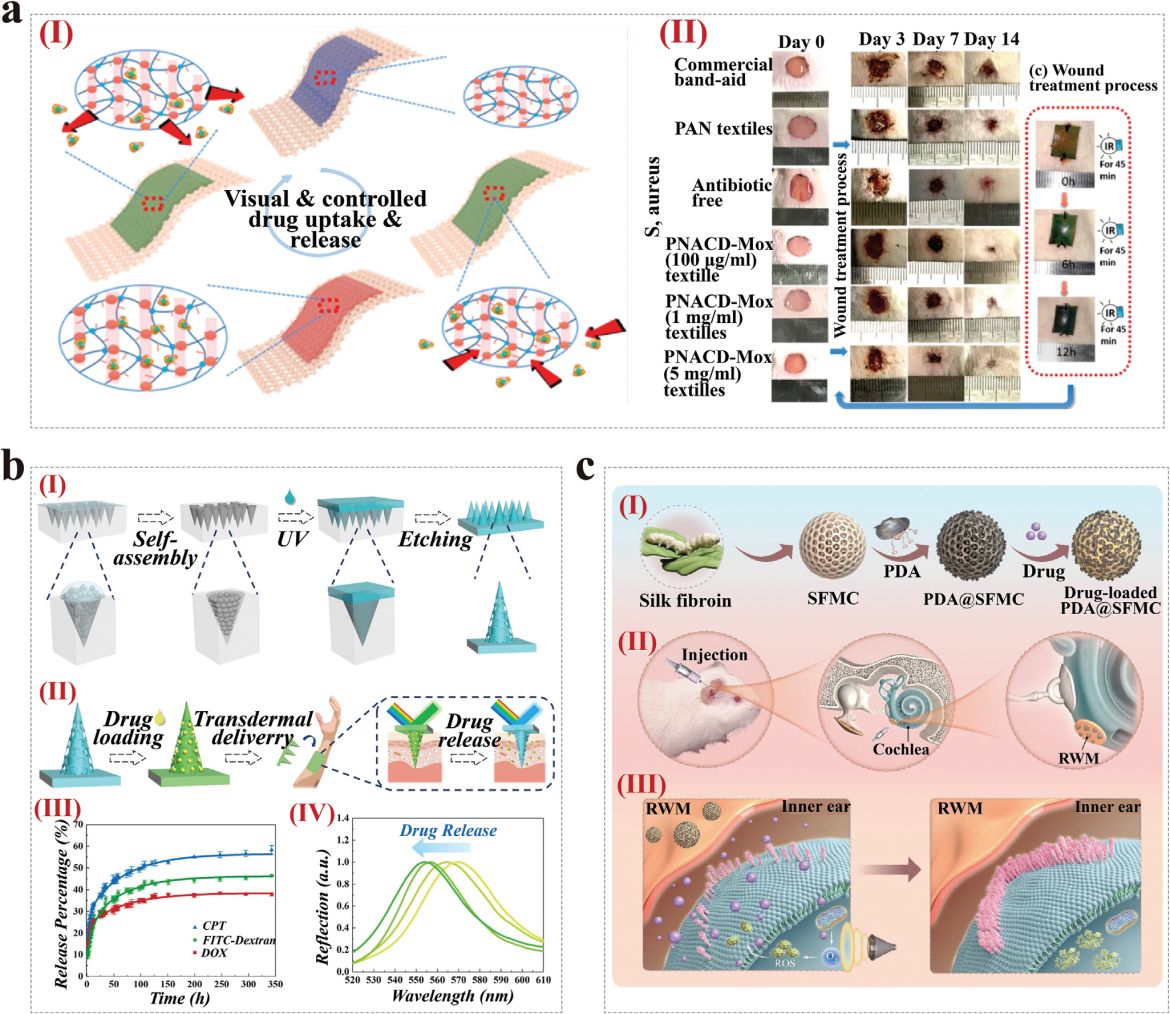
CPCs for drug delivery. a) (I) Schematic drug release of the thermochromic skin patch based on CPCs. (II) Graphs of the treatment effectiveness of wounds from 0 to 7 days with different drug carriers in markets. Reproduced with permission.^[^ ^94^ ^]^ Copyright 2020, American Chemical Society. b) Schematic diagram of the preparation flow (I) and drug delivery and release in the application of the IOMNs; (III) Release behavior of three types of drugs from IOMNs; (IV) Blue‐shift reflection peak of IOMNs during drug release. Reproduced with permission.^[^ ^95^ ^]^ Copyright 2022, Wiley‐VCH. c) (I) Schematic diagram of the multifunctional silk microcarrier preparation process. (II) Schematic representation of delivering silk microcarriers into the ear. (III) Schematic diagram of the drug across the RWM into the inner ear and its therapeutic effect. Reproduced with permission.^[^ ^96^ ^]^ Copyright 2023, The Authors, published by Wiley‐VCH.

Most current extracorporeal drug delivery systems combine fluorescent molecules with drugs to monitor drug release and diffusion. It requires external imaging equipment to detect drug release, which is inconvenient to operate and has poor sensitivity.^[^ ^139^, ^140^ ^]^ Due to their unique nanostructures and optical properties, CPCs are widely used in in vitro drug delivery systems.^[^ ^141^, ^142^ ^]^ They are capable of loading and delivering drugs in a controlled manner. Personalized administration under specific external stimuli (such as temperature, ultrasound, and low voltage current) according to the patient's condition can effectively prevent side effects of drug resistance and overdose.^[^ ^143^, ^144^ ^]^ Additionally, CPC‐based drug delivery vehicles can monitor the drug delivery process through color changes, providing real‐time insight into the patient's condition. These features suggest that CPCs have great significance in wound healing, targeted therapy, diabetes treatment, etc.^[^ ^145^, ^146^, ^147^, ^148^, ^149^ ^]^ For example, Gong et al. developed a controlled thermochromic skin patch by coupling the CPCs formed by the self‐assembly of Fe_3_O_4_ nanoparticles and drug‐carrying pNIPAM hydrogels, which allows real‐time monitoring of drug release and minimizes drug toxicity (**Figure** **8**a).^[^ ^94^ ^]^ The loading and release of drugs from this system depends on the swelling and contraction of the hydrogel which has a higher dissolution temperature than that of the skin, and the drug is administered on‐demand under mild thermal stimulation. At the same time, the lattice spacing of CPCs changed with the hydrogel expansion and contraction, which led to a change in the CPC color. Therefore, the thermal responsive drug uptake and release behavior can provide a reliable approach for real‐time monitoring of drug levels.

In addition to microarray skin patches, inverse opal microneedle can also enable intelligent delivery of drugs. For example, a multifunctional array of inverse opal microneedles (IOMNs) for drug delivery and real‐time monitoring was designed by Lu et al. (Figure 8b).^[^ ^95^ ^]^ In this work, silica nanoparticles dispersed in ethanol solvent were tightly aggregated and self‐assembled into an ordered hexagonal structure at the gas–liquid interface in the inner wall of the microneedle mold as ethanol evaporated. Subsequently, pre‐polymerized poly‐ethylene glycol diacrylate was infiltrated into interconnected nanopores, and hybrid hydrogel microneedles were prepared by ultraviolet crosslinking. Finally, the IOMNs were obtained by etching SiO_2_. Depending on the periodically arranged porous structure of the inverse opal, IOMNs displayed distinct structural colors. When the drug was released at the appropriate site, their average RI decreased with the drug release, leading to a corresponding blue shift in their characteristic spectra and thus allowing real‐time monitoring of drug release. In a mouse model of lupus erythematosus, their safety and efficacy in drug delivery were verified by delivering methotrexate, and it was found that IOMNs successfully delivered the therapeutic drugs into the body to achieve effective treatment.

Furthermore, our team developed a multifunctional silk microcarrier based on an inverse opal structure for local drug delivery and therapy in the inner ear (Figure 8c).^[^ ^96^ ^]^ The microcarriers were constructed by modifying inverse opal microspheres with PDA using a negative replication method. The obtained microcarriers had abundant porous nanostructures and dense nanochannels, which contributed to the sustained release of drugs. We also demonstrated that the microcarriers had excellent biocompatibility and strong antioxidant properties after loading the antioxidants N‐acetylcysteine at the cellular level. Due to the PDA coating modification, the microcarriers could stay on the inner ear round window membrane tightly for a long time, facilitating the precise local delivery of the loaded drugs. The results of animal experiments showed that N‐acetylcysteine‐loaded microcarriers partially restored hearing levels, reduced outer hair cell loss, and decreased synaptic damage after noise exposure in guinea pigs. In conclusion, the unique nanostructures and optical properties of CPCs enable controlled loading and delivery of drugs with real‐time monitoring, thus making them ideal scaffolds in drug delivery.

### 4.3 Cell Research

**Figure 9:**
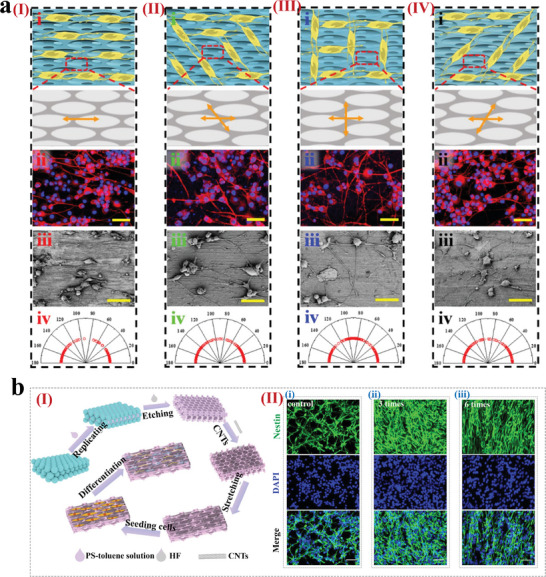
CPCs for cell culture. a) Growth of PC‐12 cells on inverse opal substrates stretched in different directions. Reproduced with permission.^[^ ^97^ ^]^ Copyright 2022, The Authors, published by Springer Nature. b) (I) Schematic diagram of carbon nanotubes‐ modified PS inverse opal film fabrication and cell culture. (II) Immunofluorescence images of neural stem cells cultured on different stretched PS inverse opal substrates. Reproduced with permission.^[^ ^98^ ^]^ Copyright 2023, The Authors, published by Elsevier.

Due to their unique micro‐nano structure and optical properties, CPCs have received widespread attention in cell research and have become candidates for “intelligent” materials. Especially in cell culture and organ‐on‐a‐chip, CPCs are highly favored. The ordered micro‐nano structure of CPCs provides an excellent substrate for cell culture, and their unique optical properties enable the detection of cell behaviors, thus, they have been widely applied in the field of organ‐on‐a‐chip. The morphology of the substrate surface has been demonstrated to effectively regulate cell adhesion, alignment, proliferation, and differentiation behaviors. CPCs possess tunable micro‐nano structures and material versatility, with the inverse opal structure's porous architecture allowing the permeation of nutrients, making it highly suitable as a cell culture substrate. For instance, Zhang et al. reported an anisotropic and conductive PS inverse opal substrate capable of inducing directional alignment of nerve cells (**Figure** **9a**).^[^ ^97^ ^]^ The cell culture substrates were prepared by filling poly(3,4‐ethylenedioxythiophene):poly(styrenesulfonate) (PEDOT:PSS) and polyacrylamide (PAAm) polymers into PS inverse opal structure. Their results indicated that stretching the PS inverse opal substrate in different directions can induce directional alignment of PC12 cells in various directions, thereby forming a complex neural network. Additionally, conductive and anisotropic inverse opal substrates were also constructed in one of our studies.^[^ ^98^ ^]^ In this work, we fabricated the substrates by combining PS inverse opal substrate and conductive carbon nanotubes (Figure 9b‐I). The inverse opal substrates exhibited good cytocompatibility and the ability to induce orientation of neural stem cells cultured on them by different degrees of stretching (Figure 9b‐II). These studies show that the stretched inverse opal substrate effectively induces cellular orientation and synaptic connectivity of nerve cells and thus has potential applications in the field of neural tissue engineering. In addition, many studies have demonstrated the potential of CPCs in other biomedical applications like organ‐on‐a‐chip by using them as a culture substrate for other types of cells.^[^ ^150^, ^151^, ^152^ ^]^

**Figure 10:**
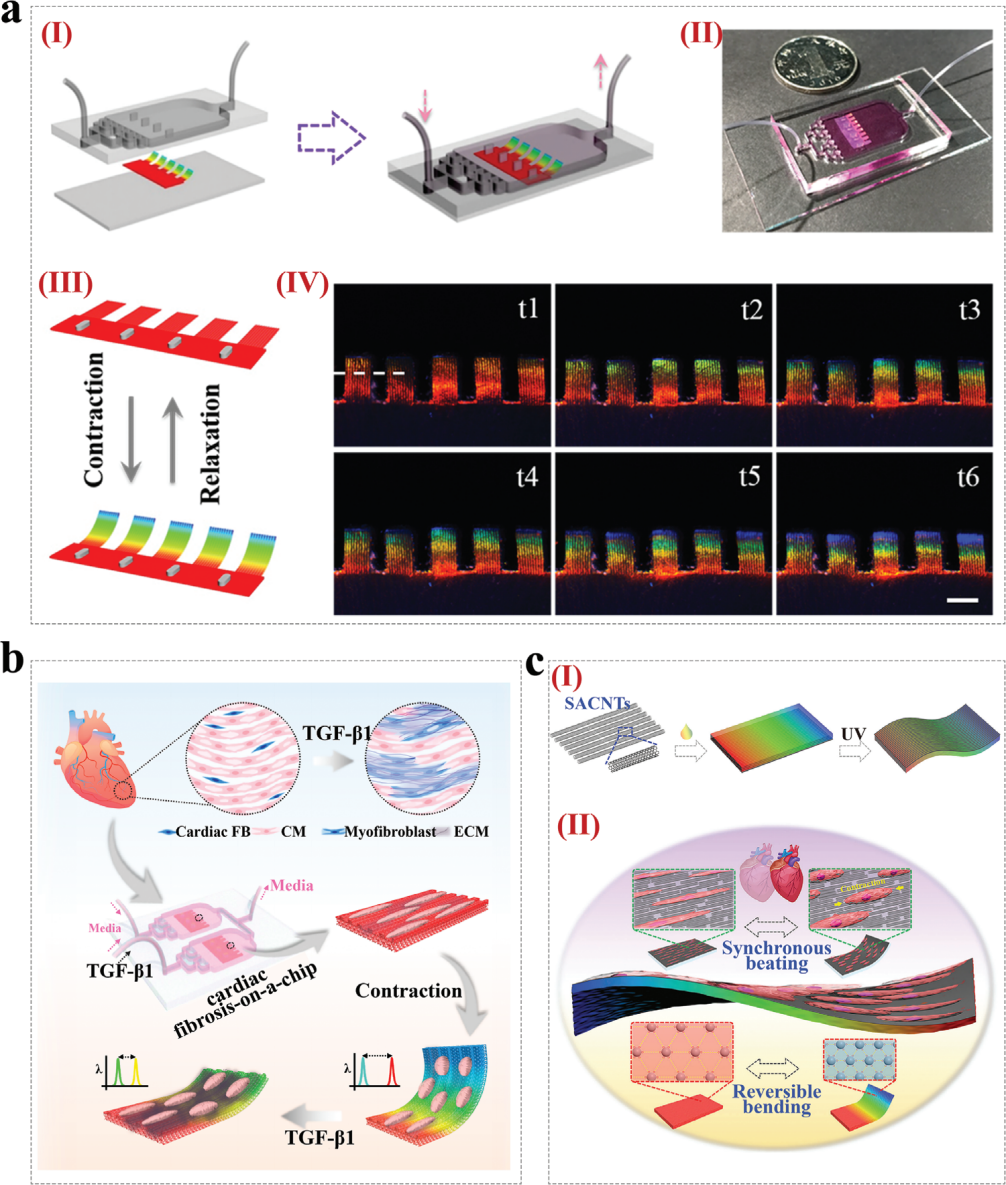
CPCs for Organ‐on‐a‐chip. a) Schema (I) and image (II) of the heart‐on‐a‐chip by combining structural color hydrogel and a microfluidic device; Schematic diagram of the structural color variation during the bent‐up process (III) and images of color changes during one myocardial cycle (IV). Reproduced with permission.^[^ ^99^ ^]^ Copyright 2018, American Association for the Advancement of Science. b) Schematic diagram of the cardiac fibrosis‐on‐a‐chip for therapy study. Reproduced with permission.^[^ ^100^ ^]^ Copyright 2024, American Chemical Society. c) Schematic diagram of the preparation of (GelMA)/SACNTs structural color hydrogel substrates (I) and their application in visualizing and fabricating heart‐on‐a‐chip (II). Reproduced with permission.^[^ ^85^ ^]^ Copyright 2022, The Authors. Published by Wiley‐VCH.

Organ‐on‐a‐chip is a microfluidic system that highly mimics the key structures and functions of human tissues and organs and is now considered a novel in vitro model capable of facilitating disease modeling and drug development, among others.^[^ ^153^, ^154^, ^155^, ^156^, ^157^, ^158^, ^159^ ^]^ The introduction of CPCs into the design and construction of organ‐on‐a‐chip can serve as an “intelligent” substrate for real‐time monitoring of the organ's physiological state and microenvironmental changes.^[^ ^160^, ^161^, ^162^, ^163^ ^]^ For example, inspired by the chameleons' structural color shift mechanism, Fu et al. fabricated a microgrooved structural color methacrylated gelatin (GelMA) hydrogel films with inverse opal nanostructure and microgrooves.^[^ ^99^ ^]^ The microgroove structure on the surface of hydrogel induced cardiomyocyte orientation, while the inverse opal nanostructure conferred the ability to visualize cardiomyocyte contraction. They combined the structural color hydrogel with microfluidics to construct a self‐reporting heart‐on‐a‐chip system (**Figure** **10a**). Additionally, Shang et al. prepared a cardiac fibrosis‐on‐a‐chip by integrating Janus structural color hydrogel into a microfluidic chip.^[^ ^100^ ^]^ The Janus structural color hydrogels with anisotropic topology were prepared by integrating microgroove structures and colloidal crystal templates. The obtained cardiac fibrosis‐on‐a‐chip provided comprehensive visuals for evaluating the treatment efficacy of stem cell therapeutic research (Figure 10b). In another study by Zhao's team, they reported a heart‐on‐a‐chip system by integrating GelMA/SACNTs substrate coated with non‐close‐packed colloidal arrays into a microfluidic device for drug evaluation, as shown in Figure 10c.^[^ ^85^ ^]^ The anisotropic surfaces of SACNTs directed the alignment of cardiomyocytes, and the good electrical conductivity of SACNTs promoted the synchronous beating of cardiomyocytes. The authors also proved the great potential of the constructed heart‐on‐a‐chip system for cardiomyocyte sensing and drug evaluation. Collectively, CPCs can be designed as various biosensors and integrated into organ‐on‐a‐chip systems to enable the detection of microenvironmental parameters and physiological indicators. Therefore, we believe that CPCs have a fascinating application prospect in the field of organ‐on‐a‐chip.

## 5 Conclusion and Perspective

This paper reviewed the classification, self‐assembly principle, and biomedical applications of CPCs. CPCs are gradually developing as an excellent optical material, making the dream of manipulating and controlling photons possible. So far, CPCs have received more and more attention, and scientists from all aspects have sought lots of ways to develop the application of CPCs. There have been a variety of photonic materials based on the photonic crystal structure of the color, photon forbidden bands, and other excellent optical properties. We provided the selection criteria for CPCs for the various applications. The applications of photonic crystals are including and not limited to biosensors, drug delivery systems, cell culture platforms, and other areas of biomedicine. In this section, we also point out the relevant areas where current CPC materials need to be improved and the future directions.

Although CPCs hold promising applications in biomedicine, some challenges still remain. First, the large‐scale manufacturing and commercialization of CPCs while maintaining consistency and quality may face technical and economic obstacles, requiring the development of efficient and low‐cost manufacturing methods. Despite the availability of well‐developed and widely commercialized nanoparticles, such as silica nanoparticles, many CPC building blocks are still very costly due to diverse modifications and remain at the lab scale.

Additionally, the biocompatibility, long‐term stability, reliability, circulation, and retention of CPCs in living organisms are crucial concerns. For example, some polymer CPCs with various chemical modifications for enhanced functions are not truly safe for in vivo applications. The preparation methods typically involve organic solvents and other toxins, and the removal of these toxins and side products is often not adequately addressed in the manufacturing process. The interaction of photonic crystal materials with biological tissues and organisms needs to be thoroughly studied to ensure their biocompatibility and non‐toxic side effects. This includes avoiding immune responses and ensuring the crystals function effectively in the complex environment of the human body.

Besides, the stability and reliability may be affected by the biological environment, such as degradation, deformation, or loss of function. Furthermore, for biomedical detection and sensing applications, the detection sensitivity and specificity of CPCs need to be further improved to ensure accurate detection and differentiation of biomarkers or disease states. Also, advanced data interpretation and analysis methods are essential for accurate and reliable interpretation of results. Finally, despite the encouraging laboratory results achieved, translating CPCs into clinical applications remains challenging, with hurdles such as regulatory approval, cost‐benefit considerations, and the complexity of clinical trials. Navigating the regulatory landscape to gain approval for biomedical applications is a complex process that involves extensive testing and validation to ensure safety and efficacy.

To address these challenges, several strategies should be considered. First, material selection for preparing CPCs should prioritize biocompatibility, stability, and optical properties. It is recommended to select materials such as silica and polymer‐based colloids that are known for their biocompatibility and nontoxicity. Also, surface modification and functionalization of the materials may be necessary to further enhance these properties and reduce the potential for immune responses while improving their specificity for biomolecular recognition, reducing nonspecific adsorption and reactions, and enhancing the specificity and stability of detection. Additionally, the design and optimization of CPC structures should also be thoroughly considered. By optimizing the size, shape, and arrangement of the crystals, their optical performance and mechanical strength can be improved. For example, inverse opals generally present stronger mechanical strength compared to close‐packed CPCs. The continuous framework of inverse opals provides a more robust structure compared to the discrete, spherical particles in close‐packed CPCs. The interconnected network of voids in inverse opals contributes to their overall structural integrity and resistance to mechanical deformation. Improvements in the fabrication process of CPCs are necessary to ensure controllability and consistency, which involves controlling manufacturing parameters, reducing defects and impurities, and improving crystal quality and stability.

In addition to improvements in materials and processes, comprehensive biocompatibility assessments should be conducted, including studies on cytotoxicity, immune response, in vivo compatibility, and other aspects, to ensure the safety and reliability of CPCs within biological systems. In addition, fostering interdisciplinary collaboration among biomedical, materials science, chemistry, and engineering disciplines to study the reliability and stability of CPCs in biomedical applications plays a pivotal role in enhancing their performance and promoting clinical translation. For example, CPCs can be integrated with smart materials and engineering technologies, such as flexible/stretchable materials, microfluidics, and wearable devices, to realize various emerging applications. Finally, establishing rigorous quality control systems and developing relevant standards and specifications are crucial to ensure that the quality and stability of CPC products meet the requirements of biomedical applications. It involves multiple basic strategies: 1) Work closely with regulatory bodies such as the FDA or other relevant organizations to align standards with regulatory requirements. 2) Create detailed SOPs for each step of the manufacturing process, from raw material selection to final product testing. 3) Collect and analyze feedback from users and customers to identify areas for improvement.

Through the continuous deepening of the aforementioned strategies, CPCs will have broader applications in biosensing, drug delivery, imaging, etc., ultimately bringing more benefits to biomedical research and clinical practice. It is worth mentioning that the biomedical application of CPCs is not limited to the aforementioned detection. Through the integrated application of mobile intelligent devices, big data, and artificial intelligence technologies, CPCs can be used for multi‐dimensional health monitoring and other medical device monitoring.

## Conflict of Interest

The authors declare no conflict of interest.
